# COVID-19 pandemic and the risk of infection in multiple sclerosis patients on disease modifying therapies: “what the bleep do we know?”

**DOI:** 10.1186/s41983-020-00177-0

**Published:** 2020-05-01

**Authors:** Salman Mansoor, Siobhan Kelly, Kevin Murphy, Aine Waters, Nauman Saleem Siddiqui

**Affiliations:** 1 Department of Neurology, Sligo University Hospital, Sligo, Ireland; 2 Division of Adult Haematology, Tufts Medical Centre, Boston, USA

**Keywords:** Coronavirus, COVID-19, Multiple sclerosis, Disease modifying therapies

## Abstract

The novel coronavirus which emerged in Wuhan province of China has taken world by surprise. Since been diagnosed in December 2019, it has been termed a “Pandemic” and there is a growing concern in physicians across the globe. As new evidence is emerging, there are various preventative strategies which are being deployed. Multiple sclerosis patients who are on disease modifying therapies (DMTs) might be at a higher risk of acquiring or a poorer outcome due to their immune status. This review looks at the available evidence in managing this global crisis.

## Background

Coronavirus (COVID-19) has been termed as a global pandemic by the World Health Organization after being first diagnosed in December 2019 (https://www.who.int/emergencies/diseases/novel-coronavirus-2019/events-as-they-happen). The number of people infected with the virus is rising at an exponential rate with data from the World Health Organization (WHO) being updated regularly (https://www.who.int/emergencies/diseases/novel-coronavirus-2019/situation-reports). The mortality rate is reported to be between 1 and 5% [1–3].

There is a growing concern in physicians across specialities as the situation is evolving. A practicing neurologist is facing a difficult clinical decision in relation to disease modifying therapies in multiple sclerosis and to the risk of COVID-19.

## Virology

The coronavirus that is linked to COVID-19 is from a family of Betacoronavirus which is from the same subgenus that caused the severe acute respiratory syndrome (SARS) virus. There is a structural similarity between the receptor-binding sites. Angiotensin-converting enzyme 2 (ACE-2), for viral entry, is the proposed binding region [4].

## Immune response in COVID-19

The cytokine response to COVID-19 yielded a rise in inflammatory cytokines (tumor necrosis factor (TNF)-α, interleukin (IL)-2R, and IL-6) [5].

Similarly, the cell response in COVID-19 patients showed a decrease in lymphocyte subsets B cells, T cells, and natural killer cells [5]. There was a lower level of helper T cells and memory helper T cells while an increased percentage of naïve helper T cells in patients who had severe disease [5]. Patients with COVID-19 also have lower level of regulatory T cells, and more obviously damaged in severe cases [5].

Both the rise in inflammatory cytokines and decrease in cell counts were related to the severity of the disease [5].

## Disease modifying therapies

There are many disease modifying treatments that are available at present with difference in their mechanism of action, as shown in Table 1.

**Table 1:** Disease modifying therapies and their probable risk categories (https://cdn.ymaws.com/www.theabn.org/resource/collection/6750BAE6-4CBC-4DDB-A684-116E03BFE634/ABN_Guidance_on_DMTs_for_MS_and_COVID19.pdf)

Disease modifying therapy	Mechanism of action	COVID-19 risk category (https://cdn.ymaws.com/www.theabn.org/resource/collection/6750BAE6-4CBC-4DDB-A684-116E03BFE634/ABN_Guidance_on_DMTs_for_MS_and_COVID19.pdf)
*Glatiramer acetate*	Shifts T cells from proinflammatory Th1 T cells to regulatory Th2 T cells reducing inflammation [6]	Safe to start or continue
Teriflunomide	Inhibiting rapidly dividing activated T cells and limited action on immune system [7]	Safe to start or continue
Interferon beta 1a, interferon beta 1b	Suppresses expression of inflammatory cytokines and increases expression of anti-inflammatory cytokines [8]	Safe to start or continue
Dimethyl fumarate	Activates NrF2 pathway, leading to increased humoral anti-inflammatory effects [9]	Safe to start or continue
Natalizumab	Monoclonal antibody that blocks T lymphocyte migration to CNS by interfering with α4β1-integrin receptor molecules on the surfaces of cells [10]	Safe to start or continue with highest efficacy
Fingolimod	Blocks release of lymphocytes acting on S1P1-5 receptor subtype [11]	Moderate risk
Alemtuzumab	Monoclonal antibody that decreases predominantly CD-52 positive B and T cells [12]	Significant risk
Cladribine	Disruption of proliferation of lymphocytes and apoptosis particularly depleting B cells (https://www.nature.com/articles/d42859-018-00029-1)	Significant risk
Ocrelizumab	Selectively targets the B lymphocytes that express the CD20 antigen triggers cell death (https://www.ocrevus.com/hcp/about/moa.html)	Significant risk
Rituximab	Selectively targets the B lymphocytes that express the CD20 antigen triggers cell death [13]	Significant risk
Siponimod	Inhibits the migration of the lymphocytes to the location of the inflammation by binding to sphingosine-1-phosphate receptor [14]	Could pose a significant risk
Ofatumumab	Antibody to anti-CD20 that inhibits early-stage B lymphocyte activation (https://www.centerwatch.com/directories/1067-fda-approved-drugs/listing/3172-arzerra-ofatumumab)]	Could pose a significant risk
Hematopoietic stem cell transplantation (HSCT)		Should be postponed

At present, the evidence is in its preliminary phase for what is safe and what might pose an increased risk for acquiring the COVID-19 infection and may lead to severe form of the disease. The Association of British Neurologists released guidelines dividing disease modifying therapies into risk groups (https://cdn.ymaws.com/www.theabn.org/resource/collection/6750BAE6-4CBC-4DDB-A684-116E03BFE634/ABN_Guidance_on_DMTs_for_MS_and_COVID19.pdf). These risk groups are shown in Table 1:

1. *Safe to start or continue*. Patients who are being started or already on interferon beta 1a, interferon beta 1b, glatiramer, teriflunomide, and dimethyl fumarate should continue it.
2. *Safe to start or continue with highest efficacy*. Natalizumab may be considered a highly effective and safe choice in the perspective of COVID-19. It may be considered in patients with high-disease activity.
3. *Moderate risk*. Fingolimod may increase the risk of acquiring COVID-19 or presenting with severe form of the disease; however, stopping it may get a rebound, so in these patients, benefits of continuing outweigh the risk. We do recommend these patients should be explained of these risks.
4. *Significant risk*. Alemtuzumab, ocrelizumab, rituximab, and Cladribine may increase the risk of acquiring and severity of COVID-19. They should be carefully considered before starting in new patients and those who are scheduled for their next infusions.
5. *Could pose a significant risk*. Ofatumumab and siponimod are not yet available in the UK or Ireland but might pose a significant risk.

### Hematopoietic stem cell transplantation

Undertaking HSCT may pose a significant risk to the patient and should be deferred at present considering the risk of serious COVID-19-related infection (https://cdn.ymaws.com/www.theabn.org/resource/collection/6750BAE6-4CBC-4DDB-A684-116E03BFE634/ABN_Guidance_on_DMTs_for_MS_and_COVID19.pdf).

### Active COVID-19 infection

In relation to active COVID-19 infection, a discontinuation or delay of any form of disease modifying therapy should be considered (https://cdn.ymaws.com/www.theabn.org/resource/collection/6750BAE6-4CBC-4DDB-A684-116E03BFE634/ABN_Guidance_on_DMTs_for_MS_and_COVID19.pdf).

## Discussion

The current recommendation by the Association of British Neurologists (ABN) lacks class 1 evidence (https://cdn.ymaws.com/www.theabn.org/resource/collection/6750BAE6-4CBC-4DDB-A684-116E03BFE634/ABN_Guidance_on_DMTs_for_MS_and_COVID19.pdf). The COVID-19 pandemic at present is expanding at an alarming rate, which may put these multiple sclerosis patients at a higher risk due to their immune status. The data from the World Health Organization (WHO) has not yet been translated in relation to this cohort of COVID-19 patients who are on disease modifying therapies. The risk estimate for the multiple sclerosis patients therefore still remains unknown.

## Recommendation

We recommend a careful consideration of disease modifying therapies in the current health crisis which is still unfolding. In relation to multiple sclerosis, we strongly urge clinicians to educate patients on disease modifying therapies and a potential risk of COVID-19. Patients should be urged to follow strict preventative measures and if possible to postpone or defer potentially high-risk disease modifying treatments.

## References

[1] "Coronavirus Age, Sex, Demographics (COVID-19) - Worldometer". www.worldometers.info.

[2] "Wuhan Coronavirus Death Rate - Worldometer". www.worldometers.info.

[3] "Report 4: Severity of 2019-novel coronavirus (nCoV)"(PDF).

[4] Zhou P, Yang XL, Wang XG, Hu B, Zhang L, Zhang W (2020). A pneumonia outbreak associated with a new coronavirus of probable bat origin. Nature..

[5] Qin C, Zhou L, Hu Z, Zhang S, Yang S, Tao Y (2020). Dysregulation of immune response in patients with COVID-19 in Wuhan.

[6] Arnon R, Sela M (1999). The chemistry of the Copaxone drug. Chem. Isr..

[7] Bar-Or A, Pachner A, Menguy-Vacheron F, Kaplan J, Wiendl H (2014). Teriflunomide and its mechanism of action in multiple sclerosis. Drugs..

[8] Kieseier BC (2011). The mechanism of action of interferon-β in relapsing multiple sclerosis. CNS Drugs..

[9] Brück J, Dringen R, Amasuno A, Pau-Charles I, Ghoreschi K (2018). A review of the mechanisms of action of dimethylfumarate in the treatment of psoriasis. Exp Dermatol..

[10] Hutchinson M (2007). Natalizumab: A new treatment for relapsing remitting multiple sclerosis. Ther Clin Risk Manag..

[11] Chun J, Hartung H (2010). Mechanism of action of oral fingolimod (FTY720) in multiple sclerosis. Clinical Neuropharmacology..

[12] Ruck T, Bittner S, Wiendl H, Meuth SG (2015). Alemtuzumab in multiple sclerosis: mechanism of action and beyond. Int J Mol Sci..

[13] Cerny T, Borisch B, Introna M, Johnson P, Rose AL (2002). Mechanism of action of rituximab. Anticancer Drugs..

[14] Behrangi N, Fischbach F, Kipp M (2019). Mechanism of siponimod: anti-inflammatory and neuroprotective mode of action. Cells.

